# The Effects of Moderate-Intensity Physical Exercise and Yoga Interventions on Stress in Hispanic College Students: A Pilot Study

**DOI:** 10.3390/sports13080266

**Published:** 2025-08-13

**Authors:** Hongxing Lu, Florentino Saenz, Preethi Raju, Ednia N. Gutierrez, Sue Anne Chew, Saraswathy Nair

**Affiliations:** Department of Health and Biomedical Sciences, University of Texas Rio Grande Valley, Brownsville, TX 78526, USA; hongxing.lu@utrgv.edu (H.L.); florentinosaenzmd@outlook.com (F.S.); preethicraju@gmail.com (P.R.); egtz1795@gmail.com (E.N.G.); sueanne.chew@utrgv.edu (S.A.C.)

**Keywords:** mental health, chronic psychological stress, acute stress responses, moderate-intensity physical exercise, yoga, stress management strategy

## Abstract

Background: Hispanic college students face high stress, which may increase their risk for chronic stress-related health issues. Effective and accessible stress management strategies for this population remain limited. Objective: This pilot study filled a gap by studying the effects of psychological stress on diet and physical activity habits and evaluating the impact of moderate-intensity physical exercise (MIPE) and yoga interventions on chronic psychological stress and acute stress responses among Hispanic college students. Methods: A total of 18 Hispanic college students participated in a 6-week intervention consisting of either MIPE or yoga, conducted twice weekly. Anthropometric measurements and lifestyle data were collected at both pre- and post-intervention timepoints. Psychological stress was evaluated using the Perceived Stress Scale-10 (PSS-10), morning salivary cortisol concentrations, and the Trier Social Stress Test (TSST). Results: Before the intervention, both perceived stress scores and morning salivary cortisol concentrations were positively correlated with the frequency of sweetened beverage intake and negatively correlated with attitudes toward exercise. Psychological stress was not significantly reduced after MIPE or yoga interventions. The salivary cortisol response to TSST was significantly improved after the yoga intervention. Conclusions: Although this pilot exploratory study suggests that short-term yoga may have beneficial effects in managing acute stress response in Hispanic college students, the efficacy of the interventions needs to be tested and replicated in a fully powered trial.

## 1. Introduction

Most college students in the United States face stress while pursuing their education. Based on the American College Health Association’s National College Health Assessment (ACHA NCHA) report from fall 2024, approximately 77.1% of undergraduate students reported experiencing moderate to high stress [1]. Furthermore, studies have indicated that university students’ perceived stress levels increase as they progress in their education (i.e., higher in senior compared to junior year) [2] and during the exam period [3]. The psychological stress caused by these factors may have an adverse impact on college students’ lifestyle and health [4,5,6,7].

Higher psychological stress can negatively impact the lifestyle of college students significantly [5,6]. It has been reported that chronic psychological stress is related to an unhealthy diet [5]. The body’s energy and nutrient requirements are raised during chronic psychological stress [8,9]. Since people may not have the time or motivation to prepare healthy and balanced food, they might take comfort in foods high in lipids, sugar, and calories but low in other nutrients [5,10]. Not only unhealthy dietary habits but also physical activity behaviors may be related to psychological stress. It has been reported that the perceived stress level is significantly negatively associated with physical activity behavior [6].

It has been demonstrated that behavior and mental health can be altered through increased stimulation of the hypothalamic–pituitary–adrenal (HPA) axis and sympathetic nervous system in response to psychological stress, resulting in an overall increase in the secretion of several hormones, including cortisol [7]. Cortisol is considered a primary stress hormone that modulates stress response and energy metabolism [7]. Under normal circumstances, cortisol’s activity and circulation levels rapidly rise in response to an acute stressor. With the removal of the stressor, the body’s cortisol levels will return to a level close to their “pre-stressed” state. If chronic stressors allow for a prolonged elevation in the levels of cortisol, the development of several psychological and physiological disorders may occur [11]. In addition, HPA axis responsiveness to an acute stressor and cortisol secretion were reduced in individuals under chronic psychological stress [12]. It has been suggested that chronic psychological stress and the consequent blunting of the HPA axis responsiveness to acute stressors may strongly affect college-age students’ health and academic performance [13,14,15]. According to the Fall 2024 ACHA NCHA report, 49.4% of college students in the United States experienced problems or challenges with their academics (37% reported stress as a factor), and in 85.3% of those reporting academic challenges, it caused moderate or high distress [1].

Several studies have investigated the different methods of managing chronic psychological stress and blocking the adverse effects of stress [16,17]. The interventions commonly implemented to reduce perceived stress include increasing physical exercise [16] and improving cognitive and emotional functioning by performing activities such as yoga [17].

Epidemiological studies have shown that physical activity has been considered a more effective method in the prevention of mental health disorders compared to placebo or relaxation and meditation interventions [16]. Yang et al. conducted a study on postpartum women in China [16]. They found that the psychological stress status in these subjects was significantly improved after three months of moderate aerobic exercise [16]. However, Koschel et al. reported that the stress levels of college students from the University of Nevada, Las Vegas, were not significantly reduced after a 3-day on-campus physical activity intervention [18]. These differences in observations of the effects of physical activity interventions on psychological stress may be due to variations in the type and duration of interventions, as well as the characteristics of the participants.

Yoga is another common stress reduction and mind–body relaxation technique that promotes mind-body balance through breathing exercises and various physical postures [17]. It is considered to have several benefits on mental health through the management of anxiety and depression, improving self-confidence, and reducing stress sensitivity [19]. Furthermore, yoga may regulate the body’s physiological system that participates in the acute stress response, reducing resting heart rate, blood pressure, and salivary cortisol levels [19].

Recent studies have shown that Hispanic students face higher stress and have less access to mental health services compared to non-Hispanic White students [20,21,22]. There is limited published research on how stress impacts the lifestyle behaviors of Hispanic college students, which can significantly influence their long-term health and academic performance [10,23]. A study conducted among US college students indicated that perceived stress was positively related to emotional eating [10]. The study did not specifically report stress-related dietary outcomes for Hispanic students, limiting the ability to draw conclusions about stress–diet interactions within this population. Additionally, a study conducted among college students in Mexico found that stress was negatively associated with mindful eating [23]. The study did not report specific associations between stress and detailed dietary habits in this population.

There is currently insufficient evidence to determine the optimal duration or frequency of physical activity and yoga interventions for college students, particularly among Hispanic populations experiencing psychological stress. Given the growing enrollment of Hispanic college students [24], it is essential to explore practical strategies for mitigating stress in this demographic. Thus, the objectives of this exploratory pilot project were to (1) study the effects of psychological stress on lifestyle (i.e., diet and physical activity habits) and (2) investigate the correlational effects of MIPE and yoga interventions on chronic psychological stress and acute stress response in Hispanic college students. In this pilot study, we hypothesized that (1) chronic psychological stress would be related to an unhealthy diet and reduced physical activity in Hispanic college students and (2) both psychological stress and the acute stress response would be improved after MIPE and yoga interventions. To the best of our knowledge, this is the first exploratory study to investigate the effects of stress management interventions on perceived stress among Hispanic college students, and South Texas Hispanic college students in particular.

## 2. Materials and Methods

### 2.1. Research Design

**Figure 1:**
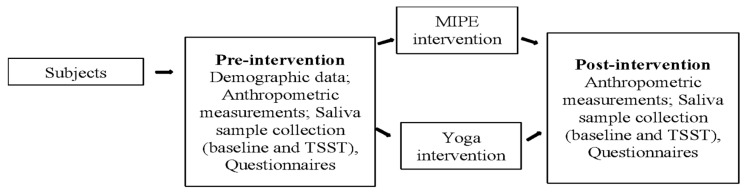
Schematic of the study. Demographic data: age, gender, health history, self-reported race, ethnicity. Anthropometric measurements: body weight, height, waist circumference, blood pressure. Saliva sample collection: baseline salivary cortisol (morning and night), PSS-10, TSST salivary cortisol (at 0, 35 min, and 50 min mark). Questionnaires: physical activity and NPAQ.

This pilot study is a quasi-intervention with a pretest–post-test design [25,26,27,28]. All the college students participated in a 6-week psychological stress management intervention program that consisted of MIPE (*n* = 10) or yoga (*n* = 8). Demographic information (i.e., age, gender, and health history) was collected from the participants during the pre-intervention visit. Before and after the interventions, the (1) physical status of the participants (i.e., body weight, height, waist circumference, and blood pressure) were measured, (2) answers to perceived stress and diet and physical activity habits surveys were collected, (3) the induced acute stress response test was conducted, and (4) the baseline (morning and night) and induced acute stress response test (i.e., pre- and post-test) saliva samples were collected to determine the salivary cortisol levels. Figure 1 is a schematic of the participants’ activities and the data collected during each activity.

### 2.2. Participants

A total of 18 college students were recruited from the Bachelor of Science in Biomedical Sciences program at the University of Texas Rio Grande Valley (UTRGV), one of the largest Hispanic-serving institutions (HSIs) in the US, where 91.2% of the student population is Hispanic [29]. All participants (*n* = 18) were between 18 and 25 years old, with a mean age of 20.17 ± 1.89 years. Around 77.8% of the subjects were female, and 22.2% were male. All subjects were self-categorized as non-exercisers, defined as individuals who had exercised less than 60 min per week for at least half a year prior to recruitment. The criteria for inclusion were US Hispanic ethnicity; 18–25 years old; no smoking or alcohol abuse; being single; the ability to read, write, and speak English; no history of diabetes, renal disease, heart disease, or hypertension; and absence of pregnancy. Before participation, the benefits and risks of this study were explained, and informed consent was obtained. This study was approved by the UTRGV Institutional Review Board (IRB Protocol #957736-4).

### 2.3. Interventions

Participants selected their intervention group (i.e., MIPE or yoga) based on personal preference to encourage motivation, comfort, and adherence to the program. The Physical Activity Guidelines for Americans indicate that adults need 150 min of moderate-intensity physical activity or 75 min of weekly muscle-strengthening training [30]. Based on our preliminary observations, some subjects felt very tired if they conducted exercise for more than one hour. Our moderate-intensity intervention is designed as a 45 min session, held twice a week (for a total of 90 min per week), accounting for 60% of a young adult’s weekly physical activity. The researchers set up the treadmill before the intervention. The MIPE intervention included a 5 min warm-up followed by 45 min of MIPE. MIPE was conducted by walking or slowly running at a speed of 3 miles/hour with a 0% incline on the treadmill in the gym, and it lasted approximately 50 min. This level and structure of activity has been validated in multiple studies as effective for reducing psychological stress and supporting metabolic health in college-aged populations [31,32]. The yoga intervention consisted of a beginner-level yoga course, taught by a certified yoga instructor, and lasted approximately 90 min. The physical activity and yoga interventions were held twice a week for six weeks at the Texas Southmost College (TSC) Recreational Center in Brownsville, TX.

During interventions, trained research staff closely monitored participants’ activity levels, physiological and psychological responses, adherence to the protocols, and recorded any adverse events. Monitoring was conducted through scheduled check-ins, self-reported activity logs, and in-person supervision to ensure compliance with the intervention protocols and to address any participant concerns. Ethical standards were maintained through a comprehensive informed consent process, assurance of participant confidentiality, and the guarantee that participants could withdraw from the study at any time without penalty.

### 2.4. Physical Measurement

General information, health history, and the participants’ informed consent forms were collected before beginning the study. Body weight in kilograms was measured using a Digital Body Weight Scale (ES-CS20M-B, Renpho, Inc., Anaheim, CA, USA). A stadiometer (Perspective Enterprises, Portage, MI, USA) was used to obtain heights in centimeters after removing shoes and wearing light clothing. An inelastic tape measure (National Center for Health Statistics, NHANES III Protocol) was used to determine waist circumference in centimeters. Body mass index (BMI) was calculated by dividing weight in kilograms by the square of the height in meters, as shown in the equation below.

$$BMI=\frac{weight\;(kg)}{height\left(m\right)*height\;(m)}$$

### 2.5. Research Instruments

#### 2.5.1. Perceived Stress Scale-10 Item

This study employed the PSS-10 questionnaire, originally developed by Cohen et al. as a psychological stress assessment instrument [33]. The PSS-10 score is considered a helpful stress predictor that is strongly related to both biological markers of stress and increased risk for stress-associated psychiatric and physical disorders. This scale includes six negatively stated and four positively stated questions, which are rated via a five-point scale that ranges from 0 to 4 (0 = never; 1 = almost never; 2 = sometimes; 3 = fairly often; 4 = very often). The total PSS score was calculated as the sum of the scores from each question, which ranged from 0 to 40, and higher scores represented higher perceived stress. In this study, PSS-10 was used to determine the chronic physiological stress level before and after the intervention.

#### 2.5.2. Trier Social Stress Test (TSST)

TSST is a protocol developed by Kirschbaum et al. in 1993 [34], which is used to induce mild stress in human research participants within a controlled environment. This test consists of three sections: public speaking, mental arithmetic, and anticipation. It is considered a powerful method to test the stress response of the HPA axis and autonomic nervous system. In this study, a modified TSST was used; it consisted of preparing and delivering an academic presentation followed by a mental arithmetic task (serial subtraction), which was given to the participants by a professional expert. Immediately before starting the TSST, the pre-TSST saliva samples were collected from the subjects, and the pre-TSST blood pressure was determined. After the collection, they were instructed to begin the 10 min study period, where they were expected to prepare a speech explaining the content of an assigned passage or article. At the 10 min mark, subjects were asked to give their prepared 5 min oral presentation to a professional expert. Participants were asked to speak for the entire 5 min and were told that their speech would be judged by a set criterion by an experienced expert. At the 15 min mark, participants perform serial subtraction as quickly and accurately as possible for 5 min. Every time a participant made a mistake, they were asked to start from the beginning. The professional expert would judge the correctness of their arithmetic task. At the 20 min mark, the post-TSST blood pressure was measured. The saliva samples after TSST were collected at both the 35 min and 50 min mark after the start of TSST.

#### 2.5.3. Nutrition and Physical Activity Questionnaire (NPAQ-A)

The Nutrition and Physical Activity Questionnaire-Adult (NPAQ-A) was used to estimate weight and body image, eating habits, weekly physical activity level, and the participant’s readiness to change these areas. The NPAQ-A survey consists of seven nutrition-based questions related to self-reported body weight, health status, and how often food, fruit, vegetables, fast food, and breakfast are consumed. The other eight questions are related to the intensity, duration, and frequency of weekly physical activity and whether the participants cared for their health [35].

### 2.6. Saliva Samples

#### 2.6.1. Saliva Sample Collection

The saliva samples from the subjects were collected with a saliva collection aid attached to a cryovial (Salimetrics, Carlsbad, CA, USA) by the passive drool method. Baseline saliva samples were collected twice a day on two different days. The baseline morning saliva samples were collected in the morning immediately after waking up, before eating and drinking. The baseline night saliva samples were collected in the evening, before sleeping. Subjects were required not to consume food or liquids 1 h before collection. The saliva samples for TSST were collected before TSST (recorded as 0 min) and at the 35 min and 50 min mark of the TSST. At least 1 mL of saliva was collected at each timepoint. The samples were stored in a refrigerator and transported to the research laboratory on ice the following day. All samples were stored at −80 °C in the research laboratory until they were analyzed.

#### 2.6.2. Salivary Cortisol Measurement

Salivary cortisol enzyme-linked immunosorbent assay (ELISA) kits (Salimetrics, State College, PA, USA) were used to determine cortisol concentration in the saliva samples. This was performed in duplicate wells (*n* = 2) using the manufacturer’s guidelines.

### 2.7. Statistical Analysis

Statistical analysis was performed using SPSS Statistics version 27.0 (SPSS, Inc., Chicago, IL, USA). The stress levels are presented as the mean ± standard error (SE). A power analysis was conducted to determine the appropriate sample size, assuming a Type I error rate (α) of 0.05, a statistical power (1–β) of 0.80, and using estimated mean and standard deviation values from the relevant literature [36]. Descriptive statistics, including frequencies, were calculated. Differences between categorical variables were analyzed via chi-square tests. The independent sample *t*-test was used to determine differences in stress values between subjects with different frequencies of diet and physical activity habits. The PSS-10 values in different weight status, dietary, and physical activity groups were computed using Analysis of Variance (ANOVA) with post hoc analyses (Scheffé’s Test). A paired *t*-test was conducted to analyze body weight status, perceived stress, salivary cortisol concentrations (morning and night), and TSST responses before and after the interventions. A univariate linear model was used to examine whether baseline morning cortisol levels moderated the effects of two interventions on the cortisol response to the Trier Social Stress Test (TSST). All statistical tests were two-tailed. The effects were considered significant at *p* < 0.05.

### 2.8. Reliability and Validation of Study

Regarding the data collection instruments, the validation and reliability of the Perceived Stress Scale-10 (PSS-10) and the Food and Physical Activity Questionnaire-Adult (NPAQ-A) have been well-documented. The PSS-10 has demonstrated strong internal consistency, with Cronbach’s alpha values typically ranging from 0.78 to 0.91 across diverse populations [37,38]. The NPAQ-A, designed by the USDA’s Expanded Food and Nutrition Education Program (EFNEP), has undergone expert review and pilot testing to ensure content validity and has shown good test–retest reliability in community-based adult populations [39]. TSST is considered the standard for HPA axis activation [34]. Trained research personnel conducted the procedure and monitored participants throughout the experiment to ensure safety and adherence to standardized protocols.

All salivary samples were analyzed via ELISA. The strategies for ensuring the reliability and consistency of results included the following: (1) all sample analyses from each subject across the study period were performed at the end of the study; (2) for each sample, we ran the test twice in the same kit to assess the consistency of the measurements; and (3) the Elisa kits’ variability was monitored by including an internal laboratory quality control sample on every kit. These control samples were run alongside the study samples to ensure that any variations in results are due to the samples themselves rather than the ELISA kits.

## 3. Results

### 3.1. Anthropometric Characteristics, Perceived Stress, and Cortisol Associations

Before the intervention, the mean PSS-10 level for the college students was 19.17 ± 1.07 (*n* = 18). We did not find a significant difference in PSS-10 levels between the different genders *(p* = 0.933). No significant associations were observed between age, BMI, or waist circumference and baseline salivary cortisol concentrations (morning and night) in the subjects for both pre- and post-intervention periods. The morning salivary cortisol levels (*n* = 18) were marginally associated with PSS-10 before the intervention (*p* = 0.083), but this association was not significant after the intervention.

### 3.2. Associations of Perceived Stress and Cortisol Levels with Dietary and Physical Activity Habits Before the Interventions

**Figure 2:**
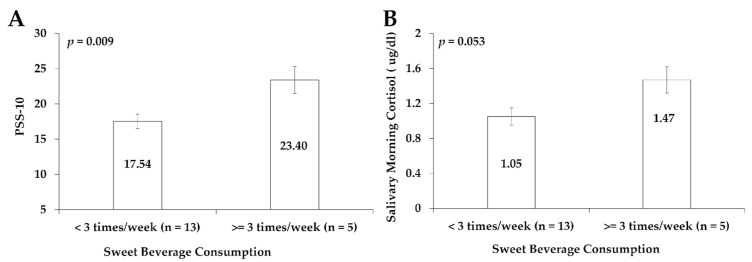
The relationships between sweet beverage consumption and PSS-10 (**A**) or salivary morning cortisol (**B**) in a total of 18 subjects before the intervention. Data are represented as mean ± standard error.

**Figure 3:**
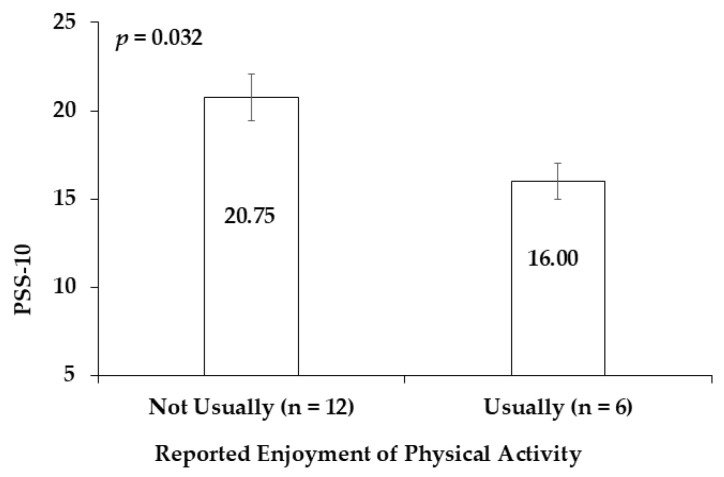
The relationships between physical activity attitude and PSS-10 in a total of 18 subjects before the intervention. Data are represented as means ± standard error.

The relationships between sweet beverage consumption or attitude towards physical activity and PSS-10 levels or morning salivary cortisol levels in the 18 subjects before the intervention are shown in Figure 2 and Figure 3. Subjects with a sweet beverage intake of three times or more a week had 33.4% (*p* = 0.009) higher PSS-10 scores (Cohen’s d = −1.558) (Figure 2A) and 40.0% (*p* = 0.053) higher morning salivary cortisol levels (Cohen’s d = −1.142) (Figure 2B), respectively, compared to subjects who consumed sweet drinks fewer than three times/week.

Power analysis indicated that a minimum of 9 participants per group (Figure 2A) and 13 participants per group (Figure 2B) would be required to detect statistically significant differences.

Before the intervention, the PSS-10 levels of subjects who reported enjoyment of physical activity were 22.89% lower than the PSS-10 levels of subjects who did not like physical activity (*p* = 0.032) (Cohen’s d = 1.178) (Figure 3). This study did not identify significant associations between nighttime salivary cortisol concentrations and lifestyle behaviors, including diet and physical activity habits.

Power analysis indicated that a minimum of 11 participants per group (Figure 3) would be required to detect statistically significant differences.

### 3.3. Physiological and Psychological Stress and Salivary Cortisol Response to TSST in the Intervention Groups

The physiological and psychological stress and stress response characteristics of subjects in the MIPE and yoga intervention groups are presented in Table 1 and Table 2, respectively. No significant differences were observed in BMI, waist circumference, baseline (morning and night), and TSST salivary cortisol levels between the physical exercise (Table 1) and yoga intervention groups (Table 2) at pre- and post-intervention. The PSS-10 level post-intervention was significantly lower in the yoga group compared to the MIPE group (*p* = 0.008); this difference was not observed pre-intervention.

After the intervention, waist circumference increased by approximately 6.87% in the MIPE group (*p* = 0.048) (Table 1). In the yoga group, the TSST salivary cortisol concentration at the 50 min mark (i.e., 50 min after beginning the TSST (0 min mark) increased by 14.41% *(p* = 0.007) compared to the value before the intervention. Additionally, the changes in TSST salivary cortisol levels between the 50 min and 0 min mark (*p* = 0.047) or between the 50 min and 35 min mark (*p* = 0.030) were significantly elevated compared to these variables before the intervention (Table 2).

The power analysis indicated that a minimum of 6 participants were required in the yoga group and 92 in the MIPE group to detect a statistically significant effect.

### 3.4. Acute Stress Response to Stressor (TSST) Pre- and Post-Intervention

**Figure 4:**
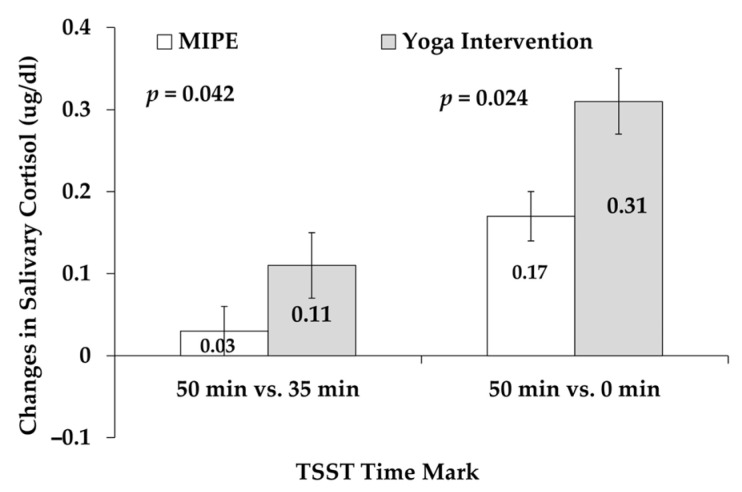
Changes in salivary cortisol concentrations in response to TSST after MIPE (*n* = 10) and yoga (*n* = 8) interventions. Data are represented as means ± standard error.

As seen in Figure 4, after the interventions, the change in cortisol level in response to the TSST in the yoga group was 82.35% (50 min vs. 0 min mark, *p* = 0.024) (Cohen’s d = −1.183) and 267% (50 min vs. 35 min mark, *p* = 0.042) (Cohen’s d= −1.020) higher compared to the MIPE group. These differences between the yoga and physical activity groups after the intervention remained statistically significant after controlling for post-study morning salivary cortisol, *p* = 0.025 (50 min vs. 0 min) and *p* = 0.044 (50 min vs. 35 min mark), respectively. These measurements were not significantly different prior to the interventions.

Based on the power analysis, a minimum of 13 participants per group would be required in both the MIPE and yoga groups to detect significant differences.

## 4. Discussion

College students face increased psychological stress during their academic studies. The American College Health Association’s National College Health Assessment reported that 26.6% of students felt they were experiencing “high stress” [1]. Neseliler et al. reported that students’ stress levels might worsen during academic exams [3]. Individuals with chronic stress may have a higher risk of facing physiological and psychological disorders [4] via several mechanisms, including modified dietary habits [5,40] and physical activity behaviors [41]. Hispanic college students face elevated psychological stress levels due to academic pressures and socioeconomic challenges compared to non-Hispanic white subjects [20,42]. Currently, to the best of our knowledge, there are no studies that have comprehensively examined the detailed patterns by which stress affects lifestyle behaviors or identified effective strategies to manage stress specifically among Hispanic college students. Therefore, the objective of this pilot study was to investigate the influence of stress on diet and physical activity habits and to examine the effects of MIPE and yoga interventions on chronic psychological stress and acute stress responses in this population. Findings from this small-scale, exploratory pilot study suggested that structured, campus-based stress management programs may help in improving student mental health and overall well-being. Even though the results in our study suggested the potential utility of yoga intervention in reducing perceived stress, its effectiveness can only be confirmed through replication in a fully powered trial. Future research should include a larger sample size to enhance statistical power and more accurately evaluate the differences and impact of the MIPE and yoga interventions.

### 4.1. Association Between Psychological Stress and Lifestyle

#### 4.1.1. Psychological Stress and Diet

This study first investigated the correlations between perceived stress or cortisol levels with dietary and physical activity habits in Hispanic college students. Our study found that stress levels increased in individuals who often consume sweet drinks. We also observed a positive correlation between salivary cortisol levels and the consumption of sweet beverages. Individuals who consumed sweet beverages three or more times a week had significantly increased PSS-10 levels and marginally higher salivary morning cortisol levels than those who drank them fewer than three times a week. These results suggest that stressed Hispanic college students have lower-nutritional-quality dietary habits, such as the consumption of high-sugar beverages. Similar results were reported in a 2021 survey study, which showed that perceived stress was positively associated with sweet intake among college students in the US [10]. A study involving 5- to 10-year-old children in Germany also found that children with higher overall daily cortisol levels consumed sweet foods more frequently than those with lower cortisol levels [43]. Cortisol is considered an appetite-stimulating hormone. It may directly stimulate the central nervous system to increase food intake and regulate the activity of other hormones (including insulin) involved in energy metabolism and appetite [44].

#### 4.1.2. Psychological Stress and Attitude to Exercise

Psychological stress influences not only diet but also the physical activity habits of individuals. The relationship between exercise habits and stress status is complex. Based on a review of the influence of stress on physical activity in 169 studies, Stults-Kolehmainen et al. observed that most research studies demonstrated that stress negatively influences physical activity [41]. For example, a study in Brazil reported that highly stressed adolescents were more likely to engage in sedentary behavior and have less time for physical exercise than individuals with lower stress levels [45]. Psychological stress affects physical activity habits via different factors and patterns [41]. Individuals under chronic stress are often associated with unhealthy diets, poor sleep, and less time for exercise [45]. Additionally, muscular recovery from high-intensity exercise may be delayed in stressed individuals [46]. These individuals may feel tired and sore, which could result in reduced exercise frequency. The hormone cortisol may be a biomarker related to psychological stress and physical activity habits [47]. A population study in Switzerland indicated that an optimal diurnal decrease in cortisol secretion was correlated with high physical activity and low sedentary behavior [47]. This pilot exploratory study did not evaluate the correlation between baseline physical activity and stress because all the subjects’ baseline physical activity levels were less than 60 min per week. To investigate the relationship between baseline physical activity and stress, we will need a range of physical activity levels in the subjects. However, we observed that the more stressed (PSS-10) the students were, the less likely they were to enjoy exercising.

### 4.2. Effects of MIPE and Yoga Intervention on Psychological Stress

We also investigated whether stress management interventions can relieve psychological stress in Hispanic college students. In our underpowered exploratory study, neither the MIPE nor the yoga intervention significantly reduced baseline cortisol concentrations measured in the morning and at night. Similarly, the mean perceived stress level did not significantly decrease following either intervention; however, a marginal reduction was observed in the yoga group. Several factors influence the effects of physical activity intervention on controlling chronic psychological stress, including the duration and frequency of the exercise program [16,48]. Therefore, findings from studies on physical activity and stress outcomes are inconsistent. For example, a study of Canadian college students found that the participants’ perceived stress worsened after a six-week high-intensity exercise program [49]. In contrast, most studies focusing on the benefits of physical activity have shown that psychological stress levels were significantly improved when the interventions lasted longer than 6 weeks, with most of these studies averaging 8 to 20 weeks [16,50]. Additionally, the frequency of exercise can significantly impact the outcome of the intervention. A systematic review and meta-analysis published in 2023, focusing on older adults, reported that physical activity sessions three times a week were effective in reducing anxiety symptoms [48]. However, the baseline stress levels significantly influence intervention outcome [51]. The mean baseline PSS-10 of our participants was more than 18, which indicates moderate baseline perceived stress, which may need more frequent interventions and ones longer than 6 weeks to show improvement. Similarly, our six-week yoga intervention may have been too short to show significant benefits in controlling chronic psychological stress. In a review article of 13 studies, the yoga interventions ranged in duration from 5 to 16 weeks (median: 12 weeks), with each session lasting between 50 and 90 min (median: 67.5 min) [52]. Our interventions were conducted during the semester and completed two weeks before the final exams, with a slow increase in stress levels due to the upcoming final exams, which could have attenuated the potential benefits of the interventions [3,53]. In the yoga group, we observed a marginal decrease in perceived stress (PSS-10), accompanied by a large effect size (Cohen’s d = 0.811). Although the reduction was not statistically significant, the observed effect size suggests a promising trend that warrants further investigation in a larger sample.

### 4.3. Effects of MIPE and Yoga Intervention on Acute Stress Response

#### 4.3.1. Effects of MIPE Intervention on Acute Stress Response

Next, we investigated whether the acute stress response to a stressor would improve after MIPE or yoga interventions. This study used the cortisol response during the TSST to indicate the activation of the HPA axis that reacts to acute stressors. The blunted or dampened acute cortisol response may represent a decreased ability to manage acute stress [54]. The published literature on the effects of exercise on cortisol response to acute stress is inconsistent [54,55,56,57]. These inconsistent observations may be due to different frequencies and intensities of exercise in these studies [54,55,56,57]. Two studies indicate that the cortisol response was significantly higher in professional athletes [56] or people with habitual physical activity [54], compared to sedentary controls. Our study did not observe significant changes in the acute salivary cortisol response to TSST stress after 6 weeks of MIPE intervention. These results are consistent with the observations from two other studies with short-term exercise programs [55,57]. The study conducted by Antoun et al. found that acute physical exercise before stressor stimulation did not improve the cortisol response [55]. Moreover, Georgakouli showed that the HPA axis activation was not significantly different after the 8-week exercise program compared to sedentary controls [57]. Therefore, the MIPE intervention may not be long enough to improve the cortisol response to an acute stressor. The small sample sizes could also have affected our observations.

#### 4.3.2. Effects of Yoga on Acute Stress Response

Although we did not find significant changes after the MIPE intervention, an improvement in cortisol response to an experimental acute stressor was observed after the yoga intervention in this pilot exploratory study. The changes in cortisol response to acute stress (TSST) after yoga were much higher than those of the MIPE intervention. Although these results cannot be interpreted broadly or conclusively due to the small-scale scope of the study, they suggest that a short-term yoga intervention may promote better stress management through a robust HPA axis response to acute stress (TSST) rather than a dampened or blunted response. A blunted HPA axis response to acute stress negatively impacts students’ academic performance [14,15]. To our knowledge, our project is the first study to explore the influences of physical activity and yoga interventions on acute stress responses in Hispanic college students. A study in 2014 by Creswell et al. also observed an improved stress response in sixty-six college students in the US after a 3-day mindfulness meditation intervention [58]. The Cresswell study compared the post-intervention cortisol change (after TSST) between the intervention and control groups. Our study was conducted on the same individuals, and the differences between pre- and post-intervention cortisol response to TSST were investigated.

Compared to physical activity (i.e., MIPE), where we did not observe an effect of the intervention, the yoga intervention improved the homeostatic acute cortisol response to stress. Our preliminary findings indicate differing effects of MIPE and yoga interventions on acute stress response in Hispanic college students. Even though we cannot draw definitive conclusions due to the underpowered, exploratory nature of this pilot study, it is worth considering the separate mechanisms by which the two interventions regulate the activity of the HPA axis. During chronic stress conditions, the HPA axis and sympathetic nervous system are overactivated, and the parasympathetic nervous system is downregulated [17]. The imbalance in functions of the central nervous system is directly linked with psychological stress-induced physiological response, including a blunted HPA axis response to acute stress [12], mental disorder (e.g., anxiety), and physiological bio-behavioral changes [17]. Physical activity regulates chronic stress and acute cortisol response through several biological pathways. After physical exercise such as MIPE, the HPA axis activity is normalized [59]. The overactivity of the HPA axis and the baseline cortisol secretion due to chronic low-level environmental stressors may be attenuated [60]. Also, the biosynthesis and activity of the endocannabinoid system are increased, which play crucial roles in exercise-induced relaxation and stress reduction [61]. Sustained longer-term exercise intervention may be required to normalize disrupted cortisol rhythms [62]. Yoga has been shown to promote the activation and function of the parasympathetic nervous system and attenuate hyperactivity of the HPA axis and sympathetic nervous system. This is partly achieved by upregulating gamma-aminobutyric acid, an inhibitory neurotransmitter, to reduce stress and anxiety [17].

It is beyond the scope of this exploratory, pilot research project to make definitive conclusions about the efficacy of MIPE or yoga intervention on the health of Hispanic college students. Future larger-scale, fully powered, and randomized control trials are necessary to replicate the findings and fully understand the differences in the impact of physical activity and yoga on perceived stress, dietary habits, active lifestyles, and acute stress responses during stressful conditions in Hispanic college students.

## 5. Study Limitations

This study has several limitations that may affect the interpretation and generalizability of the findings. One limitation is that the durations of the MIPE and yoga interventions were not equivalent. Due to limited funding, the research team was unable to hire a private yoga instructor. As a result, participants in the yoga group attended an existing 90 min weekly yoga class offered by the university over six weeks. Additionally, during the pre-study design phase, we found that more than half of the female participants reported feeling very tired and were unable to continue exercising when the duration of physical activity approached 60 min. Therefore, we set the exercise sessions to 45 min to accommodate participants’ physical capacity and improve adherence. The effectiveness of an intervention is also influenced by session length, who delivers it, and participant preferences. In this study, the yoga session was conducted by a professor-level instructor, which may have increased participants’ attention and enhanced the overall effectiveness of the intervention.

Another limitation of this study is its quasi-experimental pretest–post-test design, which was necessitated by constraints in participant availability and resources. Consequently, potential placebo effects may not have been adequately controlled for. Nevertheless, similar designs have been widely employed in prior research assessing the effectiveness of exercise and yoga interventions, particularly among diverse populations with varying ages and health conditions, even in the absence of a control group [25,26,27,28].

All participants were Hispanic undergraduate students, and both cultural characteristics and academic demands likely contributed to difficulties in recruitment, scheduling, and retention. First, familismo, a strong cultural value in Hispanic communities, may have led students to prioritize family responsibilities over attending intervention sessions [63]. Additionally, according to national data from 2020, approximately 74% of Hispanic full-time undergraduate students in the US were employed part-time while attending school [64], further limiting their availability. Second, the intervention period overlapped with a particularly demanding academic timeframe—from seven weeks to two weeks before final exams—when students are often focused on coursework and less likely to participate in extracurricular activities. These factors likely contributed to the study’s low overall retention rate (<50%), with the yoga intervention group, which involved longer session durations, experiencing the lowest retention rate at only 40%. Third, gender-related cultural norms may have also affected participation. For example, machismo may discourage Hispanic males from seeking support for stress and mental health, which could explain the low percentage of male participants (only 22%). This gender imbalance may limit the generalizability of the findings and highlight the need for culturally sensitive recruitment strategies. Future studies could improve recruitment and retention by incorporating greater scheduling flexibility, incentives, and the support of additional research staff or coordinators [65].

Lastly, the small sample size in this pilot study may increase the risk of Type II error, limiting the ability to detect significant effects or explore interaction effects between interventions. While the findings should be interpreted as preliminary rather than conclusive, the observed effect size suggests a promising trend that warrants further investigation in a more adequately powered study. These initial results provide important insights that should be replicated to derive conclusions and inform the development of future, larger-scale college-based programs aimed at supporting mental health and physiological well-being among Hispanic undergraduate students.

## 6. Conclusions

The results of this pilot study suggest that a 6-week yoga intervention did not significantly improve perceived stress levels, but a marginal reduction was observed in Hispanic college students. Furthermore, the salivary cortisol response to TSST was significantly improved after the yoga intervention. The short-term, moderate physical exercise intervention may not have been long enough to show benefits of controlling psychological stress in this population. Differences in time commitment/duration of the intervention (i.e., 90 vs. 45 min for yoga vs. MIPE) and delivery format may have influenced participant adherence and limited our ability to make direct comparisons between intervention outcomes. Thus, the observed effects of the yoga intervention may, in part, reflect variations in dosage or delivery methods rather than the effects of the intervention itself. As mentioned in the limitations, the differences observed between the two types of interventions should be interpreted with caution due to the small scale and preliminary scope of this study.

This study also demonstrated the possible influences of stress on Hispanic college students’ lifestyle (i.e., diet and physical activity habits), a group that has not been well investigated. We observed that perceived stress and morning salivary cortisol levels negatively affected dietary habits and attitude toward exercise. Our study was an underpowered, exploratory investigation and cannot support definitive conclusions regarding the efficacy of the intervention. Nonetheless, it offers a valuable starting point for future, more rigorous trials.

Future research should focus on optimizing the duration, intensity, and combination of stress management interventions, including yoga and physical exercise, to maximize benefits. Additionally, longitudinal studies are needed to explore the long-term effects of stress and intervention programs on health behaviors and outcomes in Hispanic college populations. Our current study used a non-randomized design, which carries a higher risk of bias due to participants’ self-selection into intervention groups. This limitation complicates the interpretation of results and weakens the ability to draw causal inferences regarding the intervention’s effects. To address these issues, future research should implement a randomized controlled trial with a larger sample size and an appropriate control group with duration and instructor consistency across interventions. Such a design would enhance statistical power, minimize bias, and provide more robust and reliable evidence regarding the intervention’s effectiveness, and validate these findings.

The preliminary findings from this small-scale pilot study should be viewed as exploratory observations rather than conclusions, serving primarily to inform the design and direction of future research. These observations will need to be replicated in larger, randomized controlled trials.

## Figures and Tables

**Table 1:** Descriptive characteristics of subjects in the MIPE intervention group (*n* = 10). Data is represented as mean ± standard error. Cohen’s d is provided as a measure of the estimate effect sizes.

	MIPE Intervention (*n* = 10)			
	Before	After	*p*-value	Cohen’s d
Body weight (kg)	61.50 ± 4.43	62.01 ± 4.42	0.180	−0.459
BMI	23.65 ± 1.71	23.84± 1.67	0.162	−0.476
Waist (cm)	74.39 ± 3.47	79.50 ± 3.61	0.048	−0.723
Blood pressure (mmHg)				
Systolic	111.70 ± 2.86	113.10 ±2.51	0.530	−0.206
Diastolic	70.30 ± 1.75	72.30 ± 2.44	0.474	−0.236
Stress (PSS-10)	20.90 ± 1.52	19.50 ± 1.47	0.542	0.201
Salivary cortisol (µg/dL)				
Morning	1.15 ± 0.15	1.13 ± 0.13	0.900	0.041
Night	0.52 ± 0.06	0.54 ± 0.08	0.596	−0.174
TSST-time mark				
Pre-TSST (0 min)	0.98 ± 0.09	1.19 ± 0.10	0.096	−0.588
TSST 35 min	1.14 ± 0.08	1.31 ± 0.10	0.204	−0.433
TSST 50 min	1.23 ± 0.04	1.33 ± 0.09	0.250	−0.389
TSST change				
35 min to 0 min	0.17 ± 0.07	0.12 ± 0.01	0.533	0.205
50 min to 0 min	0.25 ± 0.06	0.17 ± 0.04	0.266	0.375
50 min to 35 min	0.09 ± 0.07	0.03 ± 0.04	0.385	0.289

**Table 2:** Descriptive characteristics of subjects in the yoga intervention group (*n* = 8). Data is represented as mean ± standard error. Cohen’s d is provided as a measure of the estimate effect sizes.

	Yoga Intervention (*n* = 8)			
	Before	After	*p*-value	Cohen’s d
Body weight (kg)	78.33 ± 8.51	77.18 ± 7.77	0.320	0.378
BMI	28.97 ± 3.13	28.50 ± 2.80	0.299	0.408
Waist (cm)	86.23 ± 6.87	89.25 ± 7.03	0.173	−0.537
Blood pressure (mmHg)				
Systolic	127.50 ± 5.51	119.00 ± 2.07	0.147	0.576
Diastolic	79.13 ± 3.76	69.00 ± 2.09	0.069	0.760
Stress (PSS-10)	17.00 ± 1.17	12.63 ± 1.72	0.056	0.811
Salivary cortisol (µg/dL)				
Morning	1.19 ± 0.12	1.18 ± 0.07	0.902	0.045
Night	0.69 ± 0.11	0.68 ± 0.11	0.239	0.455
TSST time mark				
Pre-TSST (0 min)	0.98 ± 0.07	1.05 ± 0.06	0.109	−0.650
TSST 35 min	1.20 ± 0.06	1.24 ± 0.05	0.145	−0.579
TSST 50 min	1.18 ± 0.06	1.35 ± 0.03	0.007	−1.338
TSST change				
35 min vs. 0 min	0.22 ± 0.04	0.19 ± 0.03	0.598	0.195
50 min vs. 0 min	0.19 ± 0.06	0.31 ± 0.04	0.047	−0.848
50 min vs. 35 min	−0.02 ± 0.03	0.11 ± 0.03	0.030	−0.960

## Data Availability

The original contributions presented in this study are included in the Supplementary Material. Further inquiries can be directed to the corresponding author.

## Supplementary Materials

The following supporting information can be downloaded at: https://www.mdpi.com/article/10.3390/sports13080266/s1, Table S1: Body Weight Status and Blood Pressure of Subjects in Both MIPE and Yoga Intervention Groups (n = 18) Before Intervention. Data is represented as mean ± standard error. Table S2. Psychological Stress of Subjects in Both MIPE and Yoga Intervention Groups (n = 18) Before Intervention. Data is represented as mean ± standard error. Table S3. Lifestyle of Subjects in Both MIPE and Yoga Intervention Groups (n = 18) Before Intervention. Table S4. Body Weight Status and Blood Pressure of Subjects in Both MIPE and Yoga Intervention Groups (n = 18) After Intervention. Data is represented as mean ± standard error. Table S5. Psychological Stress of Subjects in Both MIPE and Yoga Intervention Groups (n = 18) After Intervention. Data is represented as mean ± standard error.

## Abbreviations

The following abbreviations are used in this manuscript.

ACHA NCHA	American College Health Association’s National College Health Assessment
BMI	Body mass index
HPA	Hypothalamic–pituitary–adrenal
IRB	Institutional Review Board
MIPE	Moderate-intensity physical exercise
NPAQ-A	Nutrition and Physical Activity Questionnaire-Adult
PSS	Perceived stress scale
TSST	Trier Social Stress Test
UTRGV	University of Texas Rio Grande Valley
