# Supplementary material for: The Effects of Moderate-Intensity Physical Exercise and Yoga Interventions on Stress in Hispanic College Students: A Pilot Study
Source: Sports (Basel). 2025 Aug 13;13(8):266. doi: 10.3390/sports13080266 (PMC12390243; doi:10.3390/sports13080266)
Supplement: Supplementary file 1 [file sports-13-00266-s001.zip › sports-3746777-supplementary.pdf]

Supplementary Materials

Table S1. Body Weight Status and Blood Pressure of Subjects in Both MIPE and Yoga Intervention Groups (*n* = 18) Before Intervention. Data is represented as mean ± standard error.

ID	group	gender	prestudy bodyweight	Height	prestudy body mass index	prestudy waist	prestudy blood pressure systolic	prestudy blood pressure diastolic
			(kg)	(m)	(kg/(m2)	(cm)	(mmHg)	(mmHg)
P2	MIPE	Female	53.80	1.56	22.11	75	100	65
P3	MIPE	Female	53.00	1.63	19.95	66	97	65
P4	MIPE	Male	67.20	1.73	22.45	76	110	65
P5	MIPE	Female	52.40	1.60	20.47	66	120	70
P6	MIPE	Female	50.40	1.59	19.94	66	109	78
P7	MIPE	Female	52.60	1.60	20.55	71	112	65
P11	MIPE	Female	67.40	1.70	23.32	75	118	73
P13	MIPE	Female	73.80	1.55	30.72	88	111	68
P16	MIPE	Female	51.00	1.56	20.96	63	112	77
P17	MIPE	Female	93.40	1.61	36.03	98	128	77
Y01	Yoga	Female	51.00	1.57	20.69	66	112	73
Y02	Yoga	Female	81.60	1.52	35.32	90	139	75
Y05	Yoga	Female	65.40	1.64	24.32	82	125	87
Y06	Yoga	Female	85.80	1.70	29.69	90	120	80
Y09	Yoga	Male	130.00	1.65	47.75	130	153	94
Y10	Yoga	Female	58.40	1.52	25.28	73	128	74
Y11	Yoga	Male	76.00	1.85	22.21	78	138	61
Y15	Yoga	Male	78.40	1.72	26.50	80	105	89

Table S2. Psychological Stress of Subjects in Both MIPE and Yoga Intervention Groups (*n* = 18) Before Intervention. Data is represented as mean ± standard error.

ID	prestudy salivary cortisol morning	prestudy salivary cortisol night	prestudy salivary cortisol TSST-0 min	prestudy salivary cortisol TSST-35min	prestudy salivary cortisol TSST-50min	prestudy TSST salivary cortisol change 35min vs 0min	prestudy TSST salivary cortisol change 50min vs 35min	prestudy TSST salivary cortisol change 50min vs 0min	prestudy perceived stress
	(ug/dl)	(ug/dl)	(ug/dl)	(ug/dl)	(ug/dl)	(ug/dl)	(ug/dl)	(ug/dl)	(PSS_10)
P2	.81	.60	.73	1.01	1.08	.28	.07	.35	21
P3	.16	.09	.42	.89	1.01	.47	.12	.59	18
P4	.89	.78	1.21	1.41	1.32	.20	-.09	.11	16
P5	1.15	.54	.89	1.04	1.23	.15	.19	.34	22
P6	1.17	.40	1.23	1.32	1.28	.09	-.04	.05	23
P7	1.23	.80	1.20	.88	1.18	-.32	.30	-.02	13
P11	1.54	.46	1.34	1.46	1.45	.12	-.01	.11	17
P13	1.54	.60	.79	.86	1.31	.07	.45	.52	27
P16	1.15	.54	1.00	1.53	1.23	.53	-.30	.23	25
P17	1.85	.40	.97	1.04	1.21	.07	.17	.24	27
Y01	.76	.13	.96	1.12	1.02	.16	-.10	.02	13
Y02	1.51	.72	1.32	1.47	1.48	.15	.01	.16	19
Y05	1.21	.76	1.07	1.32	1.10	.25	-.22	.03	20
Y06	1.43	.38	1.03	1.38	1.39	.35	.01	.36	17
Y09	1.72	0.98	.69	.98	1.03	.29	.05	.34	20.00
Y10	0.93	0.75	.78	1.11	1.21	.33	.10	.43	11.00
Y11	0.90	1.10	.90	1.10	1.10	.20	.00	.20	18.00
Y15	1.09	0.73	1.09	1.11	1.08	.02	-.03	-.01	18.00

Table S3. Lifestyle of Subjects in Both MIPE and Yoga Intervention Groups (n = 18) Before Intervention.

**ID**	**prestudy during the past 7 Days**	**prestudy during the past 7 Days**
	physical activity enjoyable	How many times drink sweet beverages
P2	Donot	0-3 times
P3	Usually	0-3 times
P4	Usually	0-3 times
P5	Donot	0-3 times
P6	Donot	0-3 times
P7	Usually	0-3 times
P11	Donot	0-3 times
P13	Donot	>3 times
P16	Donot	>3 times
P17	Donot	>3 times
Y01	Usually	0-3 times
Y02	Donot	0-3 times
Y05	Donot	0-3 times
Y06	Donot	0-3 times
Y09	Donot	>3 times
Y10	Donot	0-3 times
Y11	Usually	0-3 times
Y15	Usually	>3 times

Table S4. Body Weight Status and Blood Pressure of Subjects in Both MIPE and Yoga Intervention Groups (*n* = 18) After Intervention. Data is represented as mean ± standard error

**ID**	**poststudy Bodyweight**	**poststudy body mass index**	**poststudy waist**	**poststudy blood pressure systolic**	**poststudy blood pressure diastolic**
	(Kg)		(cm)	(mmHg)	(mmHg)
P2	53.4	21.9	75.00	94.00	65
P3	54	20.3	84.00	110.00	63
P4	69	23.1	83.82	119.00	70
P5	52.4	20.5	79.00	117.00	82
P6	50.2	19.9	67.00	112.00	69
P7	53.8	21.0	67.00	110.00	67
P11	70.1	24.3	83.20	115.00	80
P13	73.2	30.5	96.00	120.00	84
P16	51	21.0	64.00	112.00	77
P17	93	35.9	96.00	122.00	66
Y01	51	20.7	80.00	121.00	71
Y02	77.2	33.4	89.00	120.00	77
Y05	67	24.9	79.00	109.00	68
Y06	87.4	30.2	95.00	116.00	75
Y09	123	45.2	135.00	122.00	68
Y10	57.8	25.0	70.00	129.00	70
Y11	75	21.9	82.00	120.00	65
Y15	79	26.7	84.00	115.00	58

Table S5. Psychological Stress of Subjects in Both MIPE and Yoga Intervention Groups (n = 18) After Intervention. Data is represented as mean ± standard error.

**ID**	**poststudy perceived stress**	**poststudy salivary cortisol morning**	**poststudy salivary cortisol night**	**poststudy salivary cortisol TSST-0 min**	**poststudy salivary cortisol TSST-35min**	**poststudy salivary cortisol TSST-50min**	**poststudy TSST salivary cortisol change 35min vs 0min**	**poststudy TSST salivary cortisol change 50min vs 35min**	**poststudy TSST salivary cortisol change 50min vs 0min**
	PSS-10	(ug/dl)	(ug/dl)	(ug/dl)	(ug/dl)	(ug/dl)	(ug/dl)	(ug/dl)	(ug/dl)
P2	24	1.01	.60	1.24	1.32	1.44	.08	0.12	.38
P3	23	.41	.09	.62	.79	.89	.17	0.1	.28
P4	22	1.03	1.14	1.11	1.28	1.18	.17	-0.1	.17
P5	23	1.10	.54	1.18	1.27	1.27	.09	0	.09
P6	17	.72	.40	1.23	1.33	1.38	.10	0.05	.05
P7	15	1.26	.66	.89	1.04	1.13	.15	0.09	.34
P11	17	1.44	.46	1.37	1.43	1.48	.06	0.05	.11
P13	12	1.62	.60	1.38	1.52	1.49	.14	-0.03	.09
P16	26	1.73	.54	1.03	1.17	1.14	.14	-0.03	.11
P17	16	1.01	.40	1.87	1.95	1.91	.08	-0.04	.05
Y01	15	1.01	.13	1.03	1.21	1.37	.18	0.16	.34
Y02	9	1.31	.72	1.32	1.47	1.48	.15	0.01	.16
Y05	21	1.43	.76	1.14	1.29	1.39	.15	0.1	.17
Y06	10	1.27	.38	1.24	1.32	1.43	.08	0.11	.38
Y09	11	1.35	.98	.92	.98	1.23	.06	0.25	.31
Y10	10	.97	.68	.89	1.21	1.31	.32	0.1	.42
Y11	18	.87	1.10	.92	1.21	1.39	.29	0.18	.47
Y15	7	1.26	.71	.97	1.23	1.19	.26	-0.04	.21
